# Structures of the Skin Microbiome and Mycobiome Depending on Skin Sensitivity

**DOI:** 10.3390/microorganisms8071032

**Published:** 2020-07-12

**Authors:** Hye Lim Keum, Hanbyul Kim, Hye-Jin Kim, Taehun Park, Seoyung Kim, Susun An, Woo Jun Sul

**Affiliations:** 1 Department of Systems Biotechnology, Chung-Ang University, Anseong 17546, Korea; hyelim0904@gmail.com (H.L.K.); hjinkim327@gmail.com (H.-J.K.); 2 Safety and Microbiology Lab, Amorepacific Corporation R&D Center, Yongin 17074, Korea; id1star@amorepacific.com (H.K.); huny802@amorepacific.com (T.P.); sy1414@amorepacific.com (S.K.)

**Keywords:** sensitive skin, microbiome, mycobiome, perceived skin sensitivity

## Abstract

Sensitive skin (SS) syndrome is a globally widespread, self-diagnosed discomfort characterized by subjective complaints. Although the skin microbiome is considered important in skin health, the relationship between the skin microbiome and skin sensitivity is still unknown. Here, we aimed to (i) investigate whether the microbiome and mycobiome of SS are distinct from those of non-sensitive skin (NS), and (ii) define the characteristics of the skin microbiome associated with skin sensitivity. A total of 42 Korean women subjects were recruited (SS, *n* = 23; NS, *n* = 19) and the microbiome/mycobiome of their right facial cheeks were analyzed. We identified the differential microbiome and mycobiome structures between SS and NS. The mycobiome of SS was more phylogenetically diverse than that of NS. *Lactobacillus* and *Mucor racemosus* were more abundant on SS than NS, whereas *Malassezia restricta* was less abundant. Interestingly, both skin microbiome and mycobiome varied according to the perceived skin sensitivities of the subjects. This study suggests that the skin microbiome and mycobiome are associated with skin sensitivity. Accordingly, it lays the foundation for developing microbiome-based cosmetics or remedies for individuals suffering from SS syndrome.

## 1. Introduction

The sensitive skin (SS) syndrome, characterized by abnormal and unpleasant sensations, such as burning, stinging, and itching, manifests as exaggerated responses to stimuli [1]. Chemicals in cosmetics, sun exposure, and seasonal changes, which generally do not provoke irritation, can act as irritants for SS. Such a hyper-reaction can occur in seemingly healthy skin although it is sometimes accompanied by erythema [1,2]. The causes of this syndrome have been attributed to various factors [3,4]. Ponts-Giraud has described three forms of the SS: (i) high sensitivity to endogenous or exogenous factors; (ii) sensitivity to environmental factors; (iii) sensitivity to cosmetics [5]. Although skin sensitivity is not classified as a dermatological disease, it affects the well-being of many people. Studies have shown that approximately half of the world population claims to suffer from this syndrome [6,7,8,9].

Although SS is a subjective skin condition, there are several methods to determine whether a person has SS, such as sting tests using lactic acid, skin irritation tests using chemicals, and diagnosis with self-reported scales [2,9]. Sensory skin irritation, identified by the lactic acid sting test (LAST), can explain chemically induced burning, stinging, or itching sensation of SS [10]. Furthermore, patch tests on SS are more likely to respond to allergens than those on non-sensitive skin (NS) [11]. Despite extensive research on the physiological properties and etiology of SS, there is currently no precise treatment strategy. Furthermore, various exogenous and endogenous factors necessitate personalized treatment strategies [12].

Extensive research centered on the involvement of the skin microbiome in skin health has been conducted to target various skin conditions, such as atopic dermatitis, psoriasis, acne vulgaris, seborrheic dermatitis, and rosacea [13,14,15]. However, the microbial community of SS has been poorly investigated [16,17]. Accordingly, we here assessed for a correlation between the skin microbiome/mycobiome and skin sensitivity.

We recruited 42 Korean women and divided them into SS (*n* = 23) and NS (*n* = 19) groups based on the results of the 0.3% sodium lauryl sulfate (SLS) patch tests and 5% LASTs along with the information collected via a sensitivity questionnaire [18]. To investigate the correlation between the skin microbiome/mycobiome and skin sensitivity, we analyzed the bacterial and fungal communities of the facial skins (right cheek) of these subjects.

## 2. Materials and Methods

### 2.1. Subject Recruitment and Sample Preparation

All the 42 Korean women aged 22–52 years, except those with chronic skin diseases, were recruited between November and December 2017. They were divided into two skin groups, SS (*n* = 23) and NS (*n* = 19), by the following criteria: the subjects were considered to have SS if they had skin irritation scores >1.0 from 0.3% SLS patch test, skin sensory scores >0.4 from 5% LAST, and sensitivity questionnaire scores ≥2 [18] (Table S1); the remaining subjects were considered to have NS. The SLS patch test was performed on the upper back and LAST was performed on the right cheek of the face. The sensitivity questionnaire included questions that fall into the categories of basic information, skin type/problems, recognition of skin sensitivity, and quality of life (Table S2). All the subjects expressed their perceived skin sensitivity (PS) on a scale from 1 to 7. Additionally, the SS group was subjected to a survey about the causes and symptoms of SS. For the collection of microbiological samples, the subjects told not to engage in facial cleansing or use any cosmetics within 24 h before the sampling. For all subjects, sampling was conducted from a 4 cm^2^ area of the right cheek by using Catch-All sample collection swabs (Epicentre, Madison, WI, USA). With the temperature and humidity controlled at 22 (±2) °C and 50% (±10%), respectively, the right cheek of the face was swabbed approximately 50 times for ≥1 min. The collected swabs were stored at −80 °C until used for genomic DNA (gDNA) extraction. All the subjects gave their informed consent for inclusion before they participated in the study, and the study protocol was approved by the Institutional Review Board (2018-1SR-N067R).

### 2.2. Assessment of Skin Parameters

After collecting the microbial samples, the subjects washed the face and waited 30 min. Then, skin parameters were measured from the area on the left cheek that was the mirror image of the sampling site on the right cheek. Skin hydration was assessed using Corneometer^®^ CM 825 (Courage + Khazaka Electronic GmbH, Cologne, Germany) and expressed in arbitrary units. The sebum level was measured using Sebumeter^®^ SM 815 (Courage + Khazaka Electronic GmbH, Cologne, Germany) and expressed as a unique value of the device. Skin elasticity was evaluated using Cutometer^®^ (Courage + Khazaka Electronic GmbH, Cologne, Germany). Wrinkles and melanin level were assessed using Antera 3D^®^ Pro (Miravex Limited, Dublin, Ireland) and expressed in pixels.

### 2.3. Bacterial and Fungal gDNA Extraction

Bacterial and fungal gDNA extraction was simultaneously conducted from each swab by following the gram-positive bacterial cell lysate protocol of the PureLink™ Genomic DNA Mini Kit (Invitrogen, Carlsbad, CA, USA) with a bead-beating step to break down the fungal cell wall. Briefly, the head of each swab sample was excised and then transferred to a 1.5 mL screw-capped tube. Then, 400 µL of digestion buffer (20 mM Tris-HCl, pH 8.0, 2 mM EDTA, and 1.2% Triton X-100) containing lysozyme (20 mg/mL) was added. After incubating at 37 °C for 60 min, 45 µL of proteinase K (20 mg/mL) and 445 µL of PureLink™ genomic lysis/binding buffer were added. Bead-beating was performed using Mini-Beadbeater-16 (BioSpec Products, Bartlesville, OK, USA) with two 5-mm stainless steel beads (QIAGEN, Germantown, MD, USA) per tube for 1 min. The tubes were consecutively incubated on ice, at room temperature, and at 55 °C for 10, 10, and 30 min, respectively. Afterward, 445 µL of 100% ethanol was added per tube, and the samples were vigorously mixed by vortexing. Subsequent steps followed the purification protocol of the kit. Finally, extracted bacterial and fungal gDNA was eluted with 30 µL PureLink™ genomic elution buffer per tube and stored at −20 °C until use.

### 2.4. Next-Generation Sequencing of 16S rRNA Genes and ITS1 Regions

The V4–V5 regions of bacterial 16S rRNA genes were amplified using PCR with primers 518F and 927R. The primer sequences were as follows: 518F (5′-CCAGCAGCYGCGGTAAN-3′) and 927R (5′-CCGTCAATTCNTTTRAGT-3′). The fungal ITS1 regions were likewise PCR-amplified using primers 18S-F (5′-GTAAAAGTCGTAACAAGGTTTC-3′) and 5.8S-1R (5′-GTTCAAAGAYTCGATGATTCAC-3′). The following thermal cycling conditions were used: initial denaturation at 95 °C for 3 min; then 33 cycles of 95 °C for 30 s, 55 °C for 30 s, and 72 °C for 5 min; followed by a final extension at 72 °C for 5 min. The PCR products were purified using AMPure XP beads (Beckman Coulter Ltd., Wycombe, UK). Indexing PCR was performed to barcode each sample using i7 and i5 index adapters of the Illumina Nextera XT Index Kit v. 2. The thermal cycling conditions were as described above, except that only eight amplification cycles were performed. The products were purified, as indicated above. Paired-end sequencing (2 × 300 bp) was performed by Macrogen Inc. (Seoul, Korea) by using the Illumina MiSeq platform. The Illumina sequencing data were uploaded to the National Center for Biotechnology Information (NCBI) Sequence Read Archive (SRA) database under the SRA and BioProject accession numbers SRR11605054–SRR11605095 and PRJNA627788 for the microbiome data, respectively, and SRR11604870–SRR11604911 and PRJNA627798 for the mycobiome data.

### 2.5. Analysis of the Skin Microbiome and Mycobiome

Skin microbiome sequences were processed using the plugins of the QIIME™ 2 (Quantitative Insights Into Microbial Ecology) pipeline 2018.11.0 [19]. Primer sequences were removed from the bacterial sequences by using Cutadapt 1.18 [20] with the default settings. Reads that contained no bacterial primer sequence or a poor-quality primer sequence (i.e., error rate >10%) were eliminated at this step. Paired-end sequence reads were merged, and the combined reads were trimmed based on Q-score 20 by using VSEARCH [21] and the quality-filter [22] plugin. The remaining sequences were denoised using a 370 trim length via the deblur [23] plugin, whereby bacterial amplicon sequence variants (ASVs) were identified. The 99% Greengenes database was used to assign bacterial taxonomy by using the feature-classifier classify-sklearn [24] plugin, and mitochondrial or chloroplastic ASVs were eliminated. The ASVs were aligned using the phylogeny align-to-tree-mafft-fasttree plugin, and α-diversity (shannon, Faith’s phylogenetic diversity (PD), Pielou’s evenness) and β-diversity were determined with a rarefied depth of 2123 reads per sample by using the diversity plugin.

Fungal sequence reads were merged and trimmed using the ITSxpress [25] plugin with ITS1 region parameters after removing the primer sequences through the same method used for the bacterial sequences. The remaining sequences were denoised using a 180-trim length via the deblur plugin, whereby fungal ASVs were identified. The taxonomic assignment was conducted using the 99% UNITE database. Using the same methods described above, ASV alignment was conducted, and diversities were determined with a rarefied depth of 2478 reads per sample. QIIME2 default parameters were used, except for the values described above.

### 2.6. Microbial Network Analysis

To construct the microbial networks of the two skin groups, we selected bacterial and fungal ASVs with frequencies ≥50% and excluded those with an abundance of zero in each group. We selected 106 ASVs in the NS group (99 bacterial and seven fungal ASVs) and 112 ASVs in the SS group (105 bacterial and seven fungal ASVs) for further analysis. The microbial networks were estimated using the “SParse InversE Covariance estimation for Ecological Association Inference” (SpiecEasi) package and visualized with the igraph package in R.

### 2.7. Statistical Analysis

Spearman’s rank correlation coefficient was calculated to determine the correlations among the skin parameters and PS by using the Hmisc package in R. Analysis of similarity (ANOSIM) was conducted to identify the factors that differentiate the microbial community. The Wilcoxon rank-sum test, or *t*-test, was performed to determine whether the UniFrac dissimilarities, alpha diversities, and taxonomies of the two skin groups (SS and NS) were significantly different. To identify the significant gradient factors influencing or associated with the community structure of the skin microbiome and mycobiome, the factors of metadata were fitted to the ordinated space based on unweighted UniFrac dissimilarity by using the envfit function of vegan in R with 999 permutations. Linear discriminant analysis (LDA) Effect Size (LEfSe) analysis was conducted with an LDA score ≥3.0 to identify the significant ASVs in each group.

## 3. Results

### 3.1. The Skin Characteristics of the Subjects

The 42 women subjects were divided into SS (*n* = 23) and NS (*n* = 19) groups according to the skin irritation, skin sensory, and sensitivity questionnaire scores, as described in Table S1. The mean age of the subjects was 35.0 years (±8.6 years), and there was no significant difference in mean age between the SS (32.6 ± 8.2 years) and NS (37.8 ± 8.3 years) groups (Table 1, point a). The indices of the subjective questionnaire evaluations showed that PS (expressed on a scale from 1 to 7) was significantly higher (*p* < 0.001) on SS than on NS (Table 1, point b). However, no significant difference was observed in any of the assessed skin parameters between the two groups (Table 1, point c).

### 3.2. Comparison of the Microbiome and Mycobiome of SS and NS

We obtained a mean of 20,728 (microbiome) and 39,266 (mycobiome) sequence reads for the community analysis (Table S3). After denoising using deblur, 1210 and 342 unique bacterial and fungal ASVs were identified, respectively.

**Figure 1:**
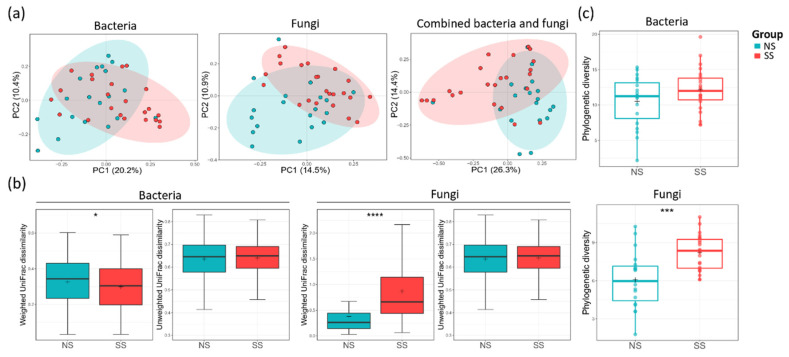
Bacterial and fungal communities of the two skin groups. (**a**) The bacterial (analysis of similarity (ANOSIM), *p* = 0.002) and fungal (ANOSIM, *p* = 0.006) principal coordinate analysis (PCoA) plots by unweighted UniFrac distance and combined bacterial/fungal PCoA by Bray–Curtis distance (ANOSIM, *p* = 0.001). (**b**) The UniFrac dissimilarity in the bacterial and fungal communities calculated within each skin group. (**c**) The alpha diversities of the bacterial and fungal communities by using Faith’s PD index. The statistical significance of the differences between the skin groups is indicated by *ns* > 0.05, * *p* ≤ 0.05, *** *p* ≤ 0.001, and **** *p* ≤ 0.0001.

Principal coordinate analysis (PCoA) plot based on unweighted UniFrac dissimilarity revealed that both bacterial (ANOSIM, *R* = 0.083, *p* = 0.020) and fungal (ANOSIM, *R* = 0.112, *p* = 0.006) communities were segregated between the two skin groups (Figure 1a). However, when weighted UniFrac dissimilarity was used, segregation was observed only in the fungal community (ANOSIM, *R* = 0.079, *p* = 0.027) (Figure S1a). The skin group was the major factor differentiating SS from NS in both microbiome and mycobiome. PS was another factor differentially affecting the fungal community (ANOSIM, *R* = 0.13, *p* = 0.016). Age group (20–30 s and 40–50 s) or perceived skin type (dry, combination, normal, or oily skin) did not have statistically significant effects in distinguishing the two skin groups in microbiome or mycobiome (Table S4). The weighted UniFrac dissimilarity of the fungal community was increased in the SS group, whereas the value of the bacterial community was decreased (Figure 1b). From these results, we found that the fungal community varied more among the subjects in the SS group than among those in the NS group, but, conversely, the bacterial community was more similar. For both bacterial and fungal communities, alpha diversity tended to be higher on SS than on NS (Figure 1c, Figure S1b). Faith’s PD of the fungal community was significantly higher on SS than on NS (*t*-test, *p* < 0.001), suggesting that SS mycobiome was phylogenetically more diverse than that of NS.

### 3.3. The Difference in Taxonomic Compostition Between SS and NS

**Figure 2:**
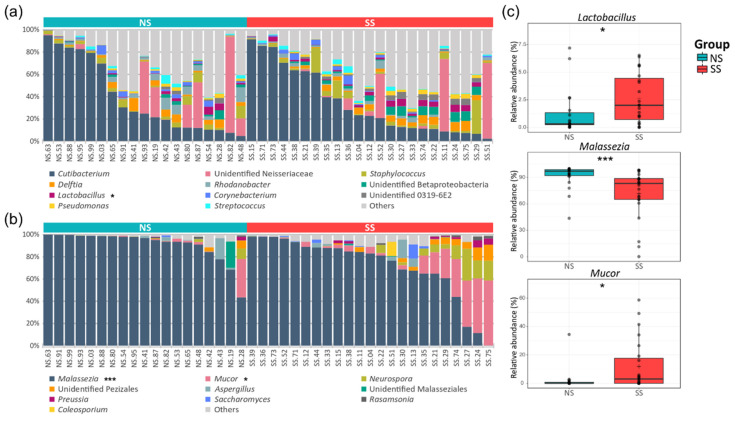
Genus level taxonomic compositions of the bacterial and fungal communities on the skin. The relative abundances of the (**a**) top 11 bacteria and (**b**) top 10 fungi in all the skin samples. (**c**) The genera with significantly different relative abundances between the two skin groups. Detailed information about the relative abundances and phylum levels are provided in Tables S5 and S6. The statistical significance of the differences between the skin groups is indicated by *ns* > 0.05, * *p* ≤ 0.05, and *** *p* ≤ 0.001.

A total of 23 phyla and 322 genera were found in the bacterial communities. Although the taxonomic composition varied depending on the subjects, the following four phyla occupied ≥90% of the skin microbiome across all the samples: Actinobacteria (39.6%), Proteobacteria (35.5%), Firmicutes (13.8%), and Bacteroidetes (4.8%). At the genus level, *Cutibacterium* (36.32%) and unidentified Neisseriaceae (10.36%) were the most abundant bacterial genera, followed by *Staphylococcus* (4.40%) and *Delftia* (3.44%). We observed a lower proportion of Actinobacteria, Proteobacteria, and Bacteroidetes, but a higher proportion of Firmicutes, on SS compared to NS. *Lactobacillus* belonging to Firmicutes was significantly more abundant on SS (Figure 2a and Table S5). In the fungal communities, seven phyla and 112 genera were identified in total. Basidiomycota (83.75%) was the predominant phylum, followed by Ascomycota (8.38%) and Mucoromycota (7.57%). Although *Malassezia* (80.38%) of the Basidiomycota phylum was the predominant fungal genus in the skin mycobiome of all the subjects, it was significantly less abundant on SS (71.61%) than on NS (91.0%). Conversely, the *Mucor* fungal genus of the Mucoromycota phylum was more abundant on SS (Figure 2b and Table S6).

**Figure 3:**
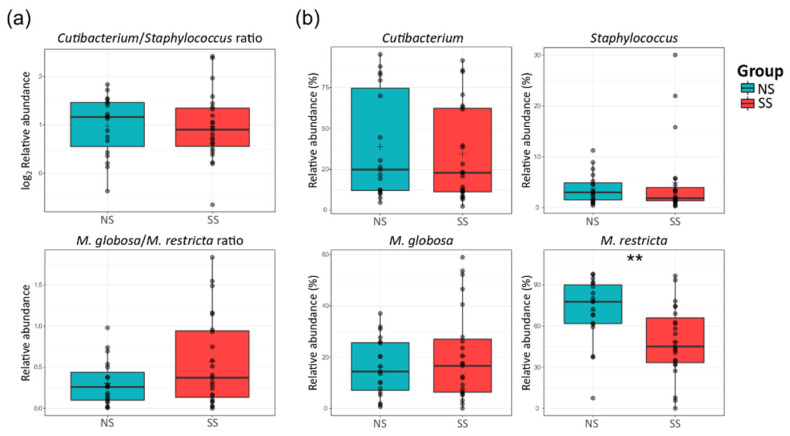
Proportions of the major bacterial genera (*Cutibacterium* and *Staphylococcus*) and fungal species (*M. globosa* and *M. restricta*) on the skin. (**a**) The ratio of *Cutibacterium* to *Staphylococcus* (log_2_ scaled relative abundance) and that of *M. globosa* to *M. restricta* (relative abundance). (**b**) Boxplots of the relative abundances of the bacterial genera *Cutibacterium* and *Staphylococcus*, and fungal species *M. globosa* and *M. restricta*. The statistical significance of the differences between the skin groups is indicated by *ns* > 0.05 and ** *p* ≤ 0.01.

There were no significant differences in the ratio of *Cutibacterium* to *Staphylococcus* or *Malassezia globosa* to *Malassezia restricta* between the two skin groups (Figure 3a). At the species level, *M. restricta* was much less abundant on SS than on NS, but *Mucor racemosus* belonging to the Mucor genus was more abundant (Figure 3b and Figure S2).

According to the LEfSe analysis results, 22 and 23 bacterial ASVs were significantly abundant on SS and NS, respectively (Figure S3). As with the results of the taxonomic composition described above, Firmicutes was significantly more abundant on SS. Deinococcus-Thermus and Verrucomicrobia phyla were significantly abundant only on SS. In the mycobiome, only the ASVs assigned to *Malassezia* spp. were found to be significantly more abundant on NS than on SS. However, the ASVs assigned to *M. racemosus* and *Phanerochaete* and those assigned to the Pezizales order of the Ascomycota phylum were more abundant on SS than on NS (Figure S4).

### 3.4. SS-Specific Microbial Network

**Figure 4:**
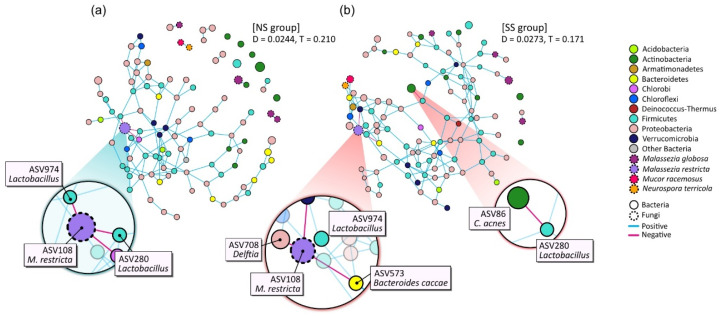
Network analysis of combined bacterial and fungal ASVs based on 50% frequency in (**a**) NS and (**b**) SS groups. Each node represents an ASV and is colored according to bacterial phylum or fungal species level. Edge color denotes whether two connected ASVs are in a positive or negative interaction. The prominent ASVs and main interactions are shown enlarged on the plot. D: density of edge; T: transitivity (clustering coefficient).

The microbial networks were constructed to identify the bacterial and/or fungal interactions by using the combined dataset pertaining to the microbiome and mycobiome (Figure 4). The overall structures of the networks noticeably differed between SS and NS. The edge density (D) of the network was higher in the SS than in the NS group, but the transitivity (T), or clustering coefficient, was substantially lower. Whereas most of the nodes in the SS microbial network participated in the interactions as one massive cluster, the NS microbial network was composed of multiple clusters, including a complex major cluster.

We also found that the main microbial interactions were different between the two skin groups (Figure 4). On NS, NS-abundant *M. restricta* (ASV108) was in negative interactions with two *Lactobacillus* ASVs (ASV974 and ASV280) and the ASV of the Chlorobi phylum (ASV855). However, in the SS group, *Delftia* (ASV708), *Bacteroides caccae* (ASV573), and the ASV of Verrucomicrobia (ASV823) replaced the negative interactions with *M. restricta* (ASV108). Additionally, on SS, *Cutibacterium acnes* (ASV86) had a negative interaction with *Lactobacillus* (ASV280).

### 3.5. Skin Microbiome and Mycobiome Associated with PS

**Figure 5:**
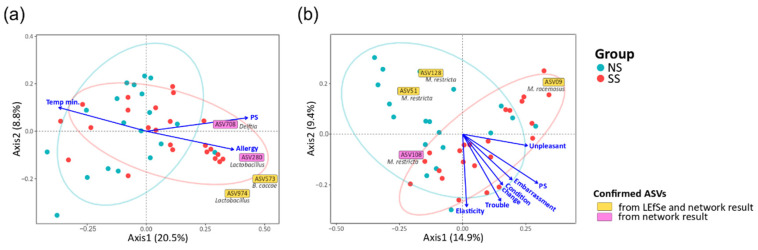
Correlation of quantitative factors with the structures of the skin (**a**) microbiome and (**b**) mycobiome. The arrows indicated the increasing directions of the quantitative gradients of the factors. Each text box represents the increasing direction of the corresponding ASV from the origin. The statistics were calculated using the envfit function of vegan in R. (Allergy, the experience of skin allergy; Temp min., minimum temperature; PS, perceived skin sensitivity; Embarrassment, embarrassment due to skin problems; Condition change, recognition of any changes in skin condition; Trouble, recognizing trouble when using cosmetics; Elasticity, skin touch elasticity; Unpleasant, unpleasant sensation on the skin).

To identify the factors in the subjective information and skin parameters (Table S2) associated with the structures of the skin microbiome and mycobiome, we fitted all quantitative factors onto the ordination space based on unweighted UniFrac via envfit R function. We found that experience of skin allergy, PS, and minimum temperature were significantly related to the bacterial community. Embarrassment due to skin problems, PS, recognition of any change in skin condition, recognizing trouble when using cosmetics, skin touch elasticity, and unpleasant sensation on skin were significantly related to the fungal community (Figure 5). Except for the factor of minimum temperature in the bacterial community, the other eight factors tended to increase toward SS, and such a tendency was more obvious in the fungal community. In particular, PS was the most explanatory variable of community structure (microbiome, *R*^2^ = 0.190, *p* = 0.016; mycobiome, *R*^2^ = 0.320, *p* = 0.002). Furthermore, the SS samples were distributed along the increasing direction of the PS axis in the PCoA ordination.

We also identified 59 bacterial ASVs (≥50% frequency in all the samples) and 11 fungal ASVs that were significantly associated with the structures of the skin microbiome and mycobiome, respectively (Figure 5 and Table S7). Regarding the skin microbiome, *Lactobacillus* (ASV974) and *B. caccae* (ASV573), confirmed from LEfSe and network results, were increased toward the SS subjects. Delftia (ASV708) and *Lactobacillus* (ASV280), which were found to be more abundant on SS according to the LEfSe result, were associated with axes of PS and experience of skin allergy. In the skin mycobiome, *M. racemosus* (ASV09) was found to be more abundant on SS according to the LEfSe result, and its population increased toward the SS subjects.

## 4. Discussion

In this study, we conducted an in-depth analysis of the skin bacterial and fungal communities on SS, whereby we revealed that there was a correlation between skin sensitivity and the skin microbiome/mycobiome. Farage et al. have summarized the research on the sensitive skin syndrome and reported that the objective physical signs of SS were lower sebum and hydration levels, and a higher skin temperature than those of NS [26]. In our data, there were no significant differences in such skin parameters between SS and NS, and there was no correlation between skin parameters and skin sensitivity (Table 1 and Figure S5). However, we found that the structures of the skin microbiome and mycobiome varied depending on perceived skin sensitivity (Figure 2). This observation suggests that the sensitive skin syndrome, which often manifests without objective physical signs, might be explained by skin microbiome/mycobiome structures.

Despite high inter-individual variability in the skin microbiome [15,27], we observed that the skin microbiome and mycobiome of SS are distinct from those of NS (Figure 1 and Figure S1). Healthier people stereotypically have been known to have greater diversity in the skin microbiome composition [28], but we found that fungal diversity was higher on SS. With the development of culture-independent sequencing, it has been shown that the *Malassezia* genus is predominant in the skin mycobiome of most people [29,30]. Our data support these previous reports, as this genus accounted for 80% of the mycobiome in all the samples in our study (Figure 2, Table S6). In the SS group, with decreasing abundance of *M. restricta*, the composition of the mycobiome became more varied among the subjects. The same tendency has been observed in other skin disorders [31,32], which follows the Anna Karenina principle (AKP) hypothesis, suggesting that alterations in the skin mycobiome contribute to their heterogeneity and are correlated with skin health [33,34].

In addition to *Malassezia*, *Cutibacterium* and *Staphylococcus* are considered as important bacterial genera in the skin microbiome. However, there were no significant differences in the relative abundances of *Cutibacterium* and *Staphylococcus* between the two skin groups (Figure 3). Hillion et al. have compared the aerobic culturable bacteria between SS and NS and reported that *Staphylococcus* is less abundant in SS patients, which differs from our results [35]. This difference in the results is presumably due to the differences between the culture-dependent and culture-independent methods used in the two studies. Interestingly in our study, the Mucor fungal genus was much more abundant on SS (Figure 2 and Table S6). Among the members of this genus, only one species (*M. racemosus*) was found in our data. This species has not received much interest in skin research. *M. racemosus* induces IgE-mediated allergic reactions and acts as a mold allergen for asthma and allergic sinusitis sufferers. *M. racemosus* has also been used as an allergen in skin-prick and provocation tests, and these sensitized individuals showed hypersensitivity in both tests [36,37,38]. Nonetheless, few studies have reported any involvement of *M. racemosus* in skin health, and our study suggests a link between this fungal species and skin sensitivity.

Furthermore, we observed cross-domain interactions in the skin microbiome and mycobiome and different patterns of these interactions by skin groups (Figure 4). On NS, *Lactobacillus* maintained a negative interaction with *M. restricta*. However, on SS, with the lower abundance of *M. restricta*, these negative interactions were shifted to *Delftia* (ASV708) and *Bacteroides caccae* (ASV573). We also observed these ASVs in the results of envfit (Figure 5 and Table S7) and LEfSe analyses (Figure S3). From the envfit results, we observed that *Delftia* (ASV708) contributed to PS, while *B.caccae* (ASV573) contributed to allergy experience. In particular, ASV280 and ASV974, assigned to the *Lactobacillus* genus, were significantly more abundant in the SS group (*p* = 0.044 and *p* = 0.008, respectively; data not shown). This observation is thought to be due to the testing method used in this study to determine the skin group. Various methods have been designed and used to identify SS, including the first developed LAST, capsaicin test, and SLS test [9,39,40]. Because some individuals may respond only to a specific stimulus, multiple testing methods should be used to precisely identify skin sensitivity. Along with the SLS test, we used the LAST, which classifies subjects with hyperactivity to lactic acid into the SS group. Lactic acid is an alpha-hydroxy acid (AHA) and has been used as an anti-aging ingredient to improve the moisture content of the skin, correct hyperpigmentation, and reduce wrinkles by increasing the production of ceramides and cell turnover rate in the epidermis. However, these acidic features can irritate the skin, leading to redness or inflammation [41]. The population of *Lactobacillus*, which produces lactic acid, might be increased on SS, with hyperactivity to lactic acid.

Our study contributes to the understanding of both the bacterial and fungal communities of SS and suggests that, in understanding skin sensitivity, the role of mycobiome is as important as the microbiome that is mainly studied in skin research. This observation may shape the designs of future studies, revealing the structure and function of the skin microbiome and mycobiome in SS. It is expected that the understanding of the microbiome/mycobiome of SS will provide the basis for the development of microbiome-based cosmetics and remedies for SS.

## Figures and Tables

**Table 1:** The skin characteristics of the subjects.

Variable	Overall (*n* = 42)	NS (*n* = 19)	SS (*n* = 23)
a. Basic Information			
Mean age (SD)	35.0 (8.6)	37.8 (8.3)	32.6 (8.2)
b. Subjective Information			
Perceived Skin Type (%)			
Dry	15 (35.7%)	6 (31.6%)	9 (39.1%)
Combination	17 (40.5%)	7 (36.8%)	10 (43.5%)
Normal	7 (16.7%)	4 (21.1%)	3 (13.0%)
Oily	3 (7.1%)	2 (10.5%)	1 (4.3%)
Perceived Skin Sensitivity (%)			
Level 1	2 (4.8%)	2 (10.5%)	0
Level 2	4 (9.5%)	4 (21.1%)	0
Level 3	6 (14.3%)	5 (26.3%)	1 (4.3%)
Level 4	9 (21.4%)	7 (36.8%)	2 (8.7%)
Level 5	12 (28.6%)	1 (5.3%)	11 (47.8%)
Level 6	7 (16.7%)	0	7 (30.4%)
Level 7	2 (4.8%)	0	2 (8.7%)
Mean (SD) ***	4.29 (1.52)	3.05 (1.13)	5.30 (0.93)
c. Skin Parameters (SD)			
Hydration	47.43 (16.80)	46.55 (16.51)	48.16 (17.37)
Sebum	17.37 (18.60)	19.68 (22.74)	15.46 (14.60)
Melanin	0.54 (0.060)	0.56 (0.049)	0.53 (0.067)
Maximum Temperature	36.49 (0.45)	36.52 (0.52)	36.46 (0.39)
Minimum Temperature	28.43 (2.57)	28.07 (2.87)	28.75 (2.30)
Average Temperature	34.20 (0.94)	34.28 (0.94)	34.13 (0.95)
Elasticity (skin touch)	69.93 (12.26)	67.53 (12.17)	71.91 (12.24)

Data are presented as mean values (SD, standard deviation) or count (%, the percentage in each group). Statistical analyses were conducted to determine the significance of the differences between the sensitive skin (SS) and non-sensitive (NS) groups for all the information and quantitative values. The statistical significance of the differences between the skin groups is indicated by *ns* > 0.05 and *** *p* ≤ 0.001.

## Supplementary Materials

The following are available online at https://www.mdpi.com/2076-2607/8/7/1032/s1, Figure S1: Bacterial and fungal communities of the two skin groups, Figure S2: Taxonomic composition of major species on the skin, Figure S3: Result of linear discriminant analysis (LDA) effect size (LEfSe) in the skin microbiome, Figure S4: Result of linear discriminant analysis (LDA) effect size (LEfSe) in the skin mycobiome, Figure S5: Correlations among the skin parameters and perceived skin sensitivity, Table S1: The criteria of the subjects selecting, Table S2: The questions of the sensitivity questionnaire used in this study, Table S3: The number of sequences reads, Table S4: The distribution of survey data using ANOSIM, Table S5: The relative abundance of bacterial communities, Table S6: The relative abundance of fungal communities, Table S7: The list of ASVs that significantly associated with skin microbiome and mycobiome.
