# Supplementary material for: Structures of the Skin Microbiome and Mycobiome Depending on Skin Sensitivity
Source: Microorganisms. 2020 Jul 12;8(7):1032. doi: 10.3390/microorganisms8071032 (PMC7409107; doi:10.3390/microorganisms8071032)
Supplement: Supplementary file 1 [file microorganisms-08-01032-s001.zip › SUPPLEMENTARY_MATERIAL_Figures.docx]

**SUPPLEMENTARY MATERIAL**

**Supplementary figure**


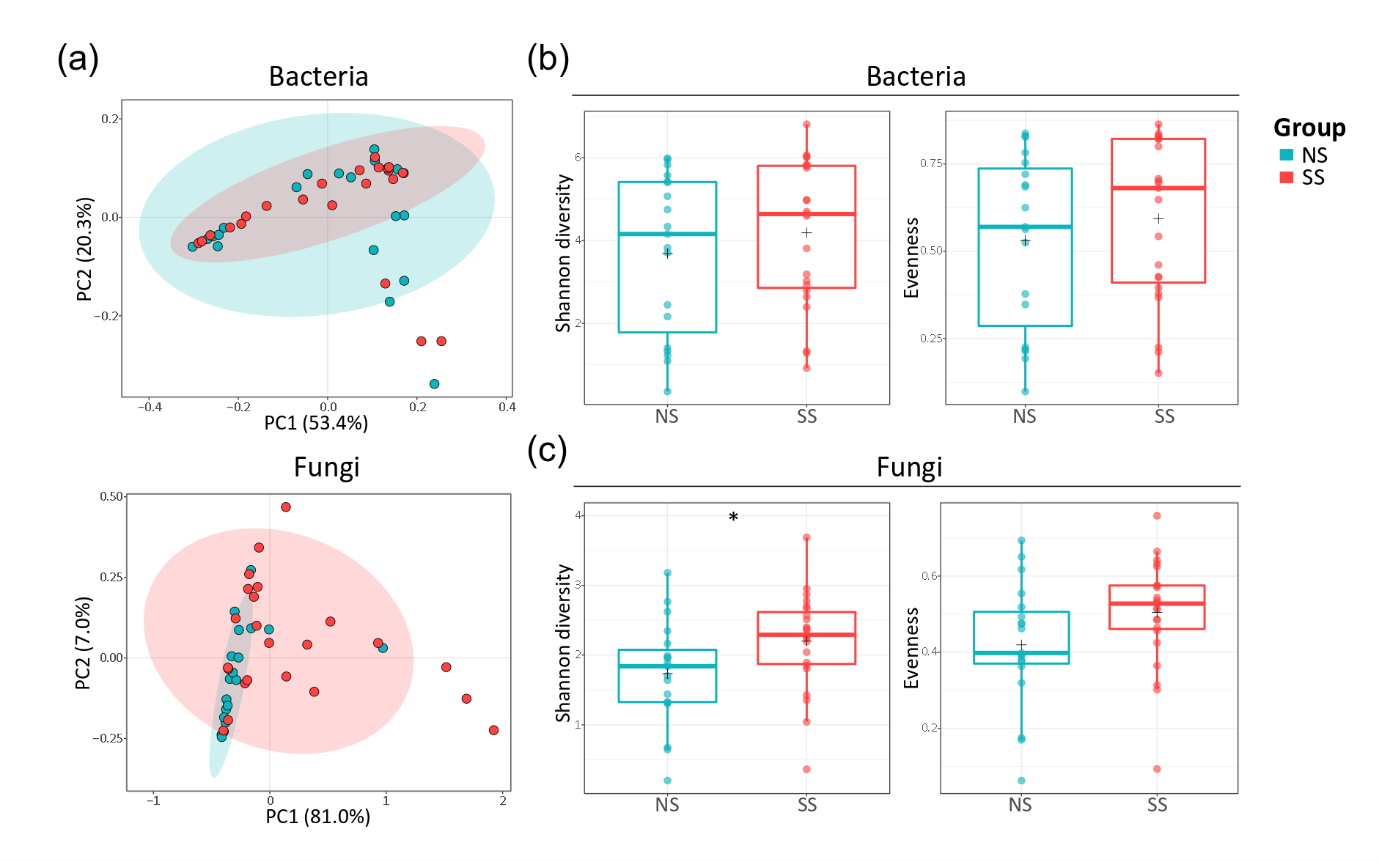


Figure S1. Bacterial and fungal communities of the two skin groups. (a) represented bacterial and fungal (ANOSIM, *P* = 0.027) PCoA based on weighted UniFrac distance. Shannon diversity and evenness were calculated in both (b) bacterial and (c) fungal communities. The statistical significance of differences between the skin groups is indicated by ns > 0.05, **P* ≤ 0.05.


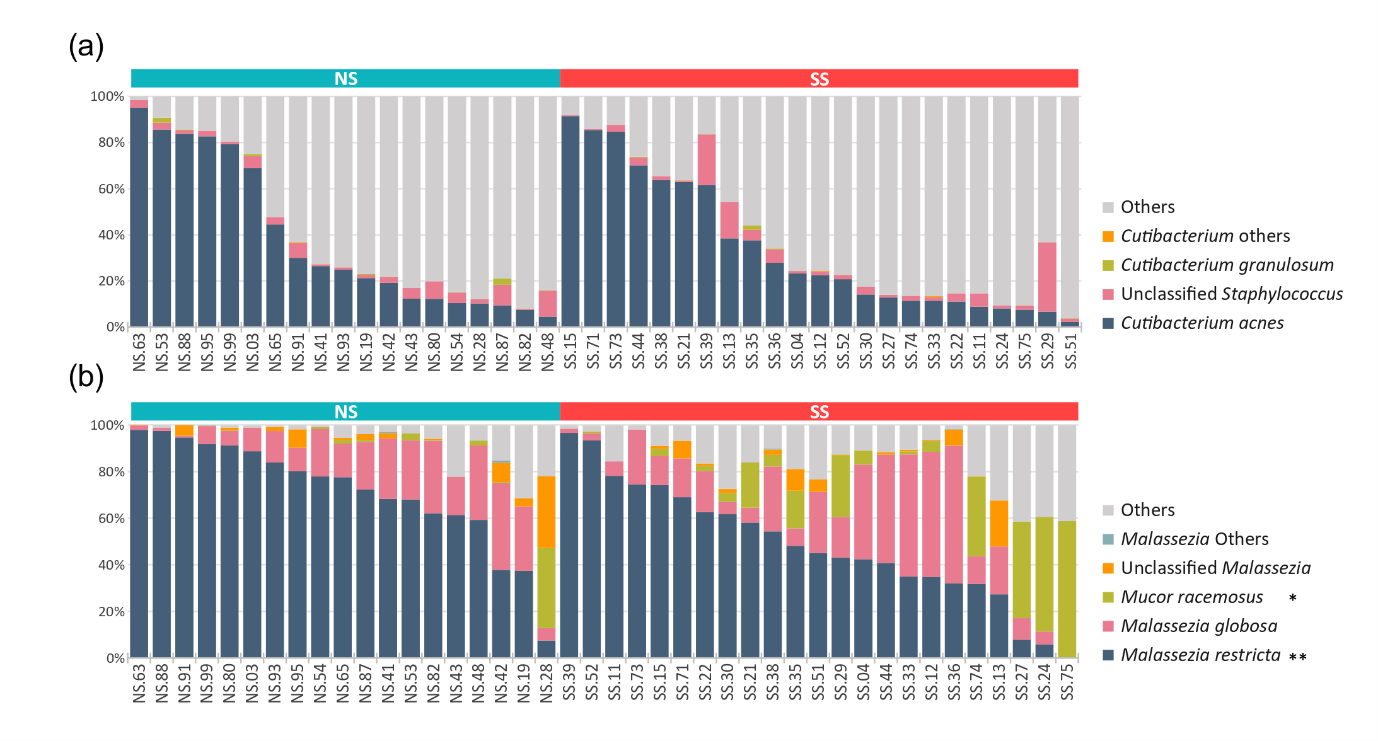


Figure S2. Taxonomic composition of major species on the skin. Barplots represented the relative abundance of (a) major bacterial species in skin microbiome and (b) fungal species in skin mycobiome. The statistical significance of differences between the skin groups is indicated by ns > 0.05, **P* ≤ 0.05, and ***P* ≤ 0.01.


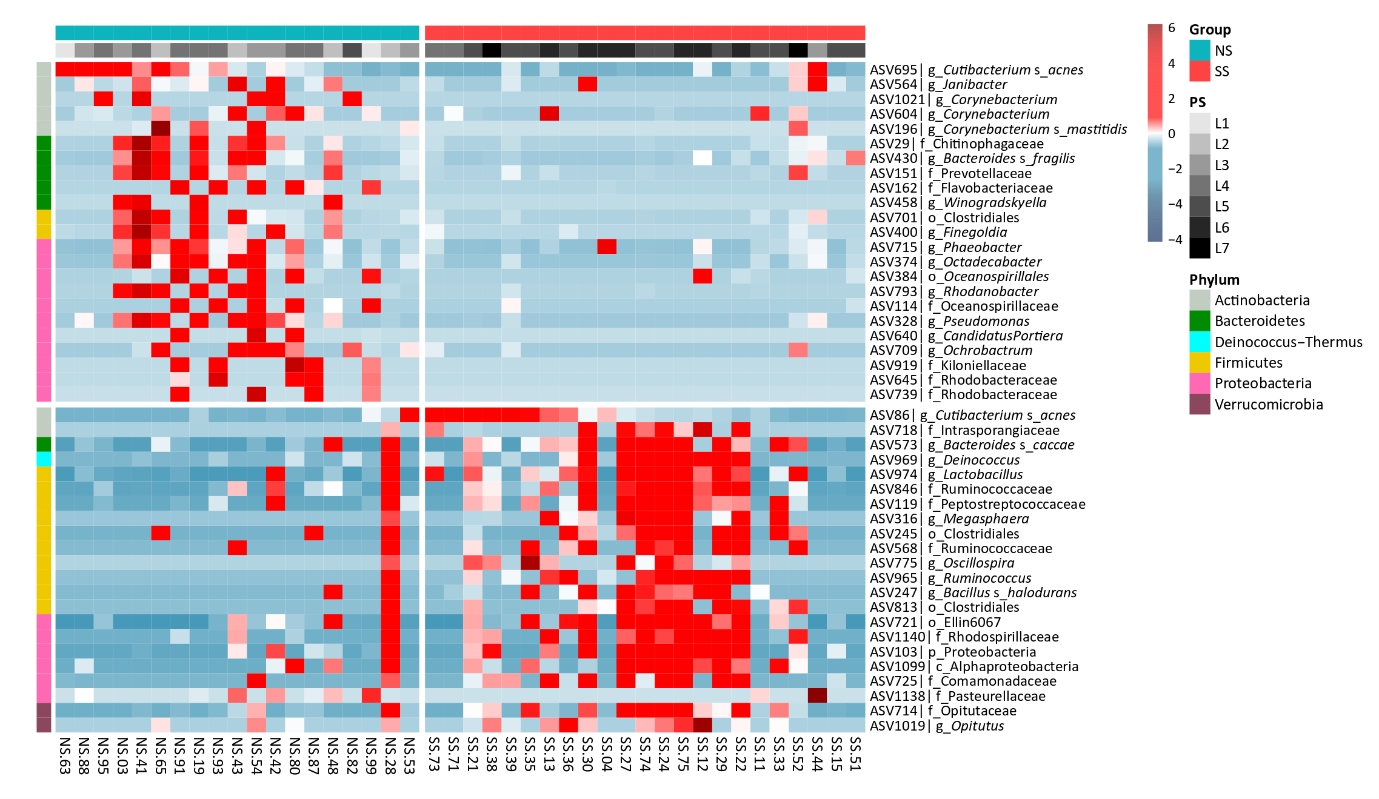


Figure S3. Result of linear discriminant analysis (LDA) effect size (LEfSe) in the skin microbiome. The heatmap was represented as a relative abundance of each ASV and scaled by each row.


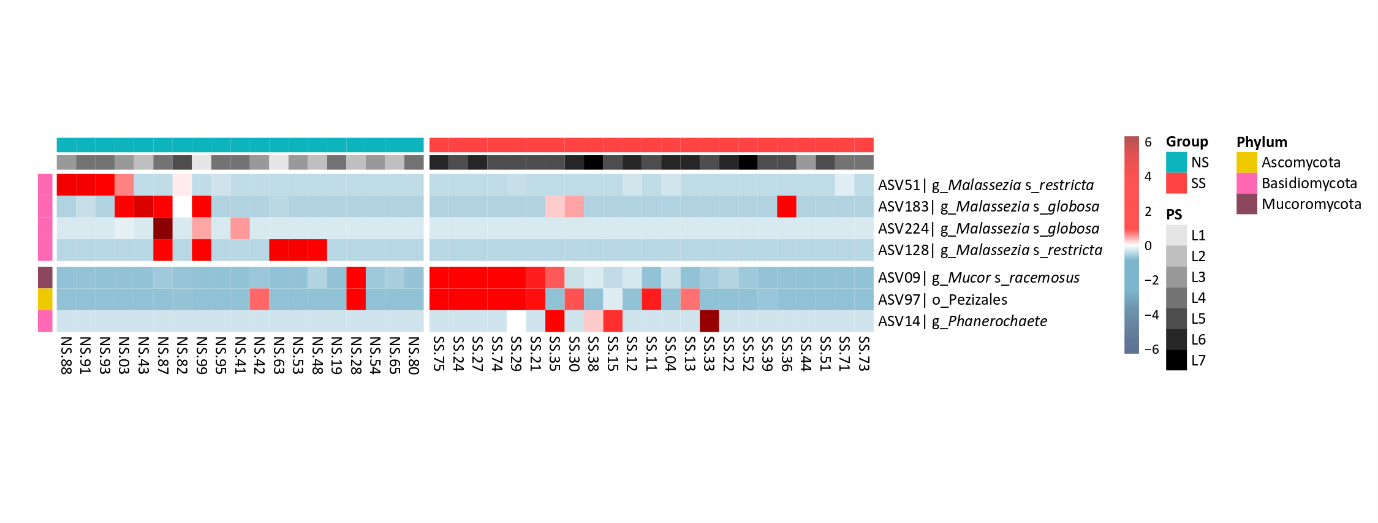


**Figure S4.** Result of linear discriminant analysis (LDA) effect size (LEfSe) in the skin mycobiome. The heatmap was represented as a relative abundance of each ASV and scaled by each row.


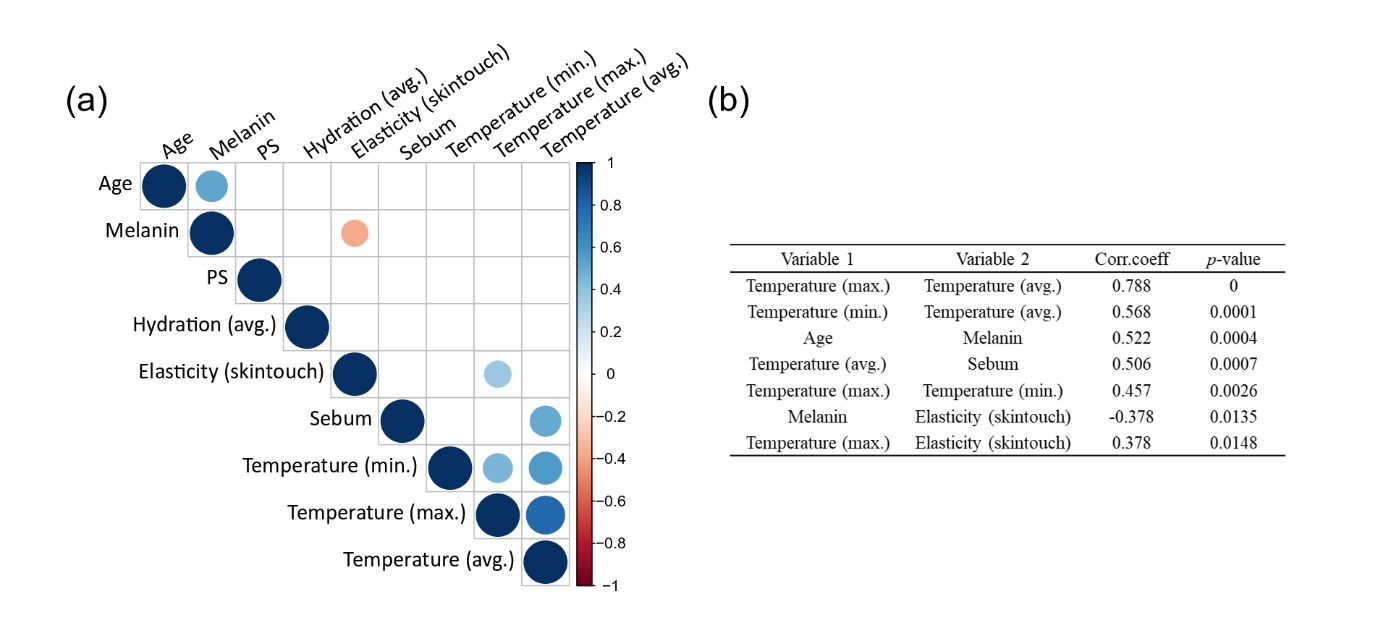


Figure S5. Correlations among the skin parameters and perceived skin sensitivity of the participants. (a) represented the correlation plot and (b) represented the statistical values between each variable.
